# Expression levels and stoichiometry of Hnf1β, Emx2, Pax8 and Hnf4α influence direct reprogramming of induced renal tubular epithelial cells

**DOI:** 10.1186/s13619-024-00202-0

**Published:** 2024-09-30

**Authors:** Xueli Hu, Jianjian Sun, Meng Wan, Bianhong Zhang, Linhui Wang, Tao P. Zhong

**Affiliations:** 1 Shanghai Key Laboratory of Regulatory Biology, Institute of Molecular Medicine, School of Life Sciences, East China Normal University, Shanghai, 200241 China; 2 Guangdong Cardiovascular Institute, Guangdong Provincial People’s Hospital, Guangdong Academy of Medical Sciences, Guangzhou, Guangdong 510100 China; 3 Department of Urology, Changhai Hospital, Shanghai, 200433 China

**Keywords:** iRECs, Bicistronic retroviral cassettes, Renal transcriptional factors

## Abstract

Generation of induced renal epithelial cells (iRECs) from fibroblasts offers great opportunities for renal disease modeling and kidney regeneration. However, the low reprogramming efficiency of the current approach to generate iRECs has hindered potential therapeutic application and regenerative approach. This could be in part attributed to heterogeneous and unbalanced expression of reprogramming factors (RFs) Hnf1β (H1), Emx2 (E), Pax8 (P), and Hnf4α (H4) in transduced fibroblasts. Here, we establish an advanced retroviral vector system that expresses H1, E, P, and H4 in high levels and distinct ratios from bicistronic transcripts separated by P2A. Mouse embryonic fibroblasts (MEFs) harboring Cdh16-Cre; mT/mG allele are utilized to conduct iREC reprogramming via directly monitoring single cell fate conversion. Three sets of bicistronic RF combinations including H1E/H4P, H1H4/EP, and H1P/H4E have been generated to induce iREC reprogramming. Each of the RF combinations gives rise to distinct H1, E, P, and H4 expression levels and different reprogramming efficiencies. The desired H1E/H4P combination that results in high expression levels of RFs with balanced stoichiometry. substantially enhances the efficiency and quality of iRECs compared with transduction of separate H1, E, P, and H4 lentiviruses. We find that H1E/H4P-induced iRECs exhibit the superior features of renal tubular epithelial cells, as evidenced by expressing renal tubular-specific genes, possessing endocytotic arrogation activity and assembling into tubules along decellularized kidney scaffolds. This study establishes H1E/H4P cassette as a valuable platform for future iREC studies and regenerative medicine.

**Supplementary Information:**

The online version contains supplementary material available at 10.1186/s13619-024-00202-0.

## Background

The generation of induced renal epithelial cells (iRECs) from fibroblasts holds great promise for regenerative medicine in renal disease (Kaminski et al. 2016, 2017; Allison 2017; Wyatt and Dubois 2017). Previous study reported that repetitive transduction of separate H1, E, P, and H4 lentiviruses convert mouse and human fibroblasts into iRECs in vitro (Kaminski et al. 2016). However, the inefficient iREC generation has become a major hurdle in the field in deciphering renal reprogramming mechanisms, modeling kidney diseases and directing iREC reprogramming in vivo (Allison 2017; Wyatt and Dubois 2017). The current renal reprogramming approach suffers from heterogeneous, weak and uncontrollable expression of RFs. This requires an advanced vector system for a high and controllable expression in fibroblasts to improve reprogramming efficiency.

*Hnf1β*, *Emx2*, *Pax8*, and *Hnf4α* are four transcriptional factors residing at the top of transcriptional hierarchy in renal gene regulatory networks (Miyamoto et al. 1997; Heliot et al. 2013; Paces-Fessy et al. 2012; Desgrange et al. 2017; Naylor et al. 2013; Bouchard et al. 2002; Narlis et al. 2007; Marable et al. 2020). During the initiation of nephrogenesis, *Hnf1β* controls proximal-intermediate nephron segment identity through regulating Notch signaling components (*Lfng*, *Dll1* and *Jag1*) and *Irx1/2* (Heliot et al. 2013). *Hnf4α* participates in the differentiation of Cdh6-expressing progenitors into proximal tubular epithelial cells (Marable et al. 2020). *Pax8* interacts with *Pax2* to regulate nephron differentiation and kidney branching morphogenesis (Narlis et al. 2007). Interestingly, *Hnf4α* is also essential for conversion of fibroblasts to functional hepatocytes (Huang et al. 2014). During organogenesis, faithful execution of developmental programs for tissue patterning and organ formation require a precise dosage and temporal expression of regulatory transcriptional factors (Miyamoto et al. 1997; Heliot et al. 2013; Paces-Fessy et al. 2012; Desgrange et al. 2017; Naylor et al. 2013; Bouchard et al. 2002; Narlis et al. 2007; Marable et al. 2020). Disruption of this delicate balance is likely to compromise tissue specification, patterning and cell reprogramming (Liu et al. 2022; Sun et al. 2019; Grand et al. 2023). For instance, the absence of HNF1β in nephron progenitors of the metanephric mesenchyme disrupts the interaction of Notch signaling and *Irx1/2*, resulting in defects in nephron differentiation, and proximal and distal tubule development (Heliot et al. 2013). In generation of induced cardiomyocytes (iCMs), the balanced expression of three transcriptional factors *Gata4* (G), *Mef2c* (M) and *Tbx5* (T) contributes to the improvement of iCM induction (Wang et al. 2015). Among six polycistronic combinations of M, G, T, the desired MGT combination that results in higher expression levels of Mef2c with lower protein levels of Gata4 and Tbx5 substantially increases iCM reprogramming efficiency, suggesting that the balanced ratios and stoichiometry of reprogramming factors profoundly influence the efficiency and quality of iCM reprogramming.

In this study, we describe the generation of a set of retroviral vectors expressing H1, E, P, and H4 from bicistronic transcripts separated by P2A sequences with antibiotics puromycin and blasticidin S selection. Each set of bicistronic combinations gives rise to distinct H1, E, P, and H4 expression levels and significantly different reprogramming efficiencies. TdTomato/mGFP fluorescent reporter mice harboring Cre recombinase under the control of the renal-specific Cadherin 16 promoter are employed to isolate MEFs for conducting iREC reprogramming. The desirable H1E/H4P cassette with the high and balanced RF expression results in a substantial improvement in iREC induction and quality, suggesting that both expression levels and stoichiometry of RFs influence efficiency and quality of iRECs. These findings represent a significant technical advance toward renal tubular epithelial cell reprogramming.

## Results

### Retroviral bicistronic RF cassettes enhance direct renal epithelial reprogramming

**Fig. 1:**
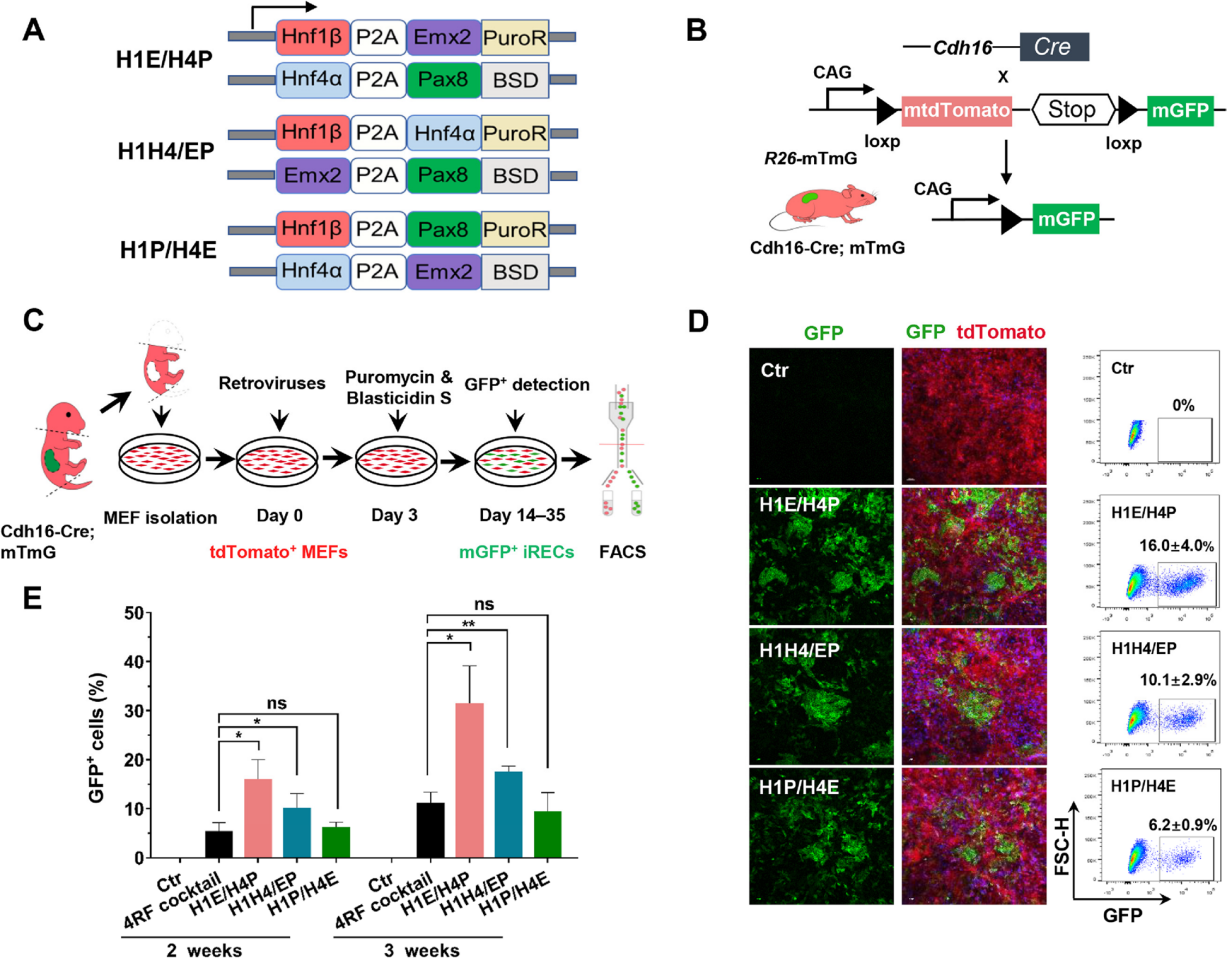
Retroviral bicistronic RF combinations enhance direct renal epithelial Reprogramming. **A** Diagram of the 6 retroviral bicistronic vectors with H1, E, H4, and P in different splicing orders separated by P2A sequence and tagged with PuroR and BSD. **B** Schematic figure showing the mating strategy for the generation of Cdh16-Cre; mTmG mice. **C** Experimental procedure for generation iRECs using 3 sets of dual bicistronic RF cassettes (H1E/H4P, H1H4/EP, and H1P/H4E). **D** Confocal images exhibiting MEFs transduced by lacZα-control (referred to as Ctr), H1E/H4P, H1H4/EP, or H1P/H4E cassettes, and FACS analyses showing the Cdh16-GFP^+^ cells after 2 weeks of culture. The experiments were conducted in three times. Data represents mean ± SD. **E** Bar charts showing quantification of percentages of Cdh16-GFP^+^ cells at 2 weeks and 3 weeks of culture by transduction of 4RF cocktail, H1E/H4P, H1H4/EP, and H1P/H4E. *n* = 3 samples. Data represents mean ± SD, **P < 0.01, ***P < 0.001, ****P < 0.0001, Student’s *t*-test. Scale bars, 100 µm (**D**)

Previous study reported that transduction of H1, E, P, and H4 from multiple lentiviruses (4RF cocktail) can induce the conversion of fibroblasts to renal tubular epithelial cells, but their reprogramming efficiencies were relatively low (Kaminski et al. 2016). Moreover, these 4 RFs are too large to be accommodated into a single viral vector due to packaging limitation of the viral particle (Kumar et al. 2001). To overcome the heterogeneous and uncontrollable expression of RFs in fibroblasts, we designed and constructed bicistronic units (Fig. 1A), including H1-P2A-E (H1E), H4-P2A-P (H4P), H1-P2A-H4 (H1H4), E-P2A-P (EP), H1-P2A-P (H1P), and H4-P2A-E (H4E). These units were separated by a P2A peptide cleavage site and tagged with puromycin N-acetyltransferase (PuroR) or blasticidin S deaminase (BSD), which conferred resistance to puromycin or blasticidin S respectively (Fig. 1A), ensuring the selection of MEFs containing 4 RFs. 3 sets of dual bicistronic RF cassettes, including H1E + H4P (H1E/H4P), H1H4 + EP (H1H4/EP), and H1P + H4E (H1P/H4E) were generated (Fig. 1A). Hnf1β (H1) was placed in the first position of bicistronic constructs due to its indispensable role for kidney development (Desgrange et al. 2017; Naylor et al. 2013). We chose retrovirus instead of lentivirus to package these bicistronic units because of their inherent features in infecting dividing cells (i.e., fibroblasts), but not differentiated and nondividing cells. These features have been demonstrated in various cell transdifferentiation systems, including iCMs and induced neuronal cells reprogramming (Qian et al. 2012; Guo et al. 2014; Warnock et al. 2011). Furthermore, mice harboring Cre-recombinase under the control of the renal epithelium-specific Cadherin-16 promoter (Cdh16-Cre) were utilized to cross to mice with tdTomato^flox/flox^ **-**mGFP dual fluorescent reporter (Shao et al. 2002). In the resulting mice (Cdh16-Cre; mT/mG), Cre-mediated recombination induces the expression of membrane GFP specifically in renal tubular epithelial cells, whereas MEFs maintain red fluorescence (Fig. 1B). TdTomato^+^ MEFs could be thus easily isolated from Cdh16-Cre; mT/mG mice to perform iREC reprogramming via directly monitoring single cell fate conversion.

To assess the amounts of retroviruses to infect MEFs in reprogramming experiments, we measured concentrations and titers of bicistronic RF retroviruses using a Retro-X qRT-PCR Titration Kit and MEF infection methods with a series of diluted viruses (Fig. S1A). We next determined the optimal amounts of retroviruses for infections by assessing multiplicity of infection (MOI) (Fig. S1B). The results showed that infecting 4 × 10^4^ MEFs with 10 µl retroviruses at 1 × 10^8^ p.f.u/ml resulted in the maximal transduction efficiency (93.2%), and the corresponding MOI value was calculated as 25 p.f.u./cell (Fig. S1B). Therefore, we utilized bicistronic RF retroviruses at the optimal MOI value of 25 to reprogram MEFs to iRECs. TdTomato^+^ MEFs were isolated from Cdh16-Cre; mT/mG mice and infected with 3 sets of bicistronic retroviruses encoding H1E/H4P, H1H4/EP or H1P/H4E. Transduced MEFs that have converted to Cdh16-expressing renal epithelial cells induces Cre-mediated loxP recombination resulting in replacement of tdTomato with mGFP (Fig. 1B). We performed fluorescence-activated cell sorting (FACS) analyses to examine mGFP expression in tdTomato^+^ MEFs to determine iREC reprogramming efficiencies (Fig. 1C). After 2 weeks of culture, bicistronic RF combinations H1E/H4P and H1H4/EP produced much higher percentages of mGFP^+^ cells than transduction of 4RF cocktail (H1E/H4P, H1H4/EP, and 4RF cocktail: 16.0 ± 4.0%, 10.1 ± 2.9%, and 5.4 ± 1.7% respectively) (Fig. 1D, E; Fig. S2A; Table S1) (Kaminski et al. 2016). Remarkably, after 3 weeks of culture, the percentage of mGFP^+^ cells was increased to almost 31% in MEFs transduced with H1E/H4P combination, 18% in MEFs with H1H4/EP transduction, almost 3- and 1.6-fold increase compared with separate H1, E, P, and H4 infections (11.2 ± 2.0%) (Fig. 1E, Fig. S2B; Table S1). However, H1P/H4E transduction generated the comparable reprogramming efficiencies compared to 4RF cocktail after 2-week and 3-week culture time (Fig. 1E). Notably, transduction of all three sets of bicistronic cassettes induced the expression of endogenous *Hnf1β*,* Emx2*,* Pax8*, and *Hnf4α* after 3 weeks of culture (Fig. S2C), suggesting a stable conversion of iRECs to renal epithelial cell fates.

**Fig. 2:**
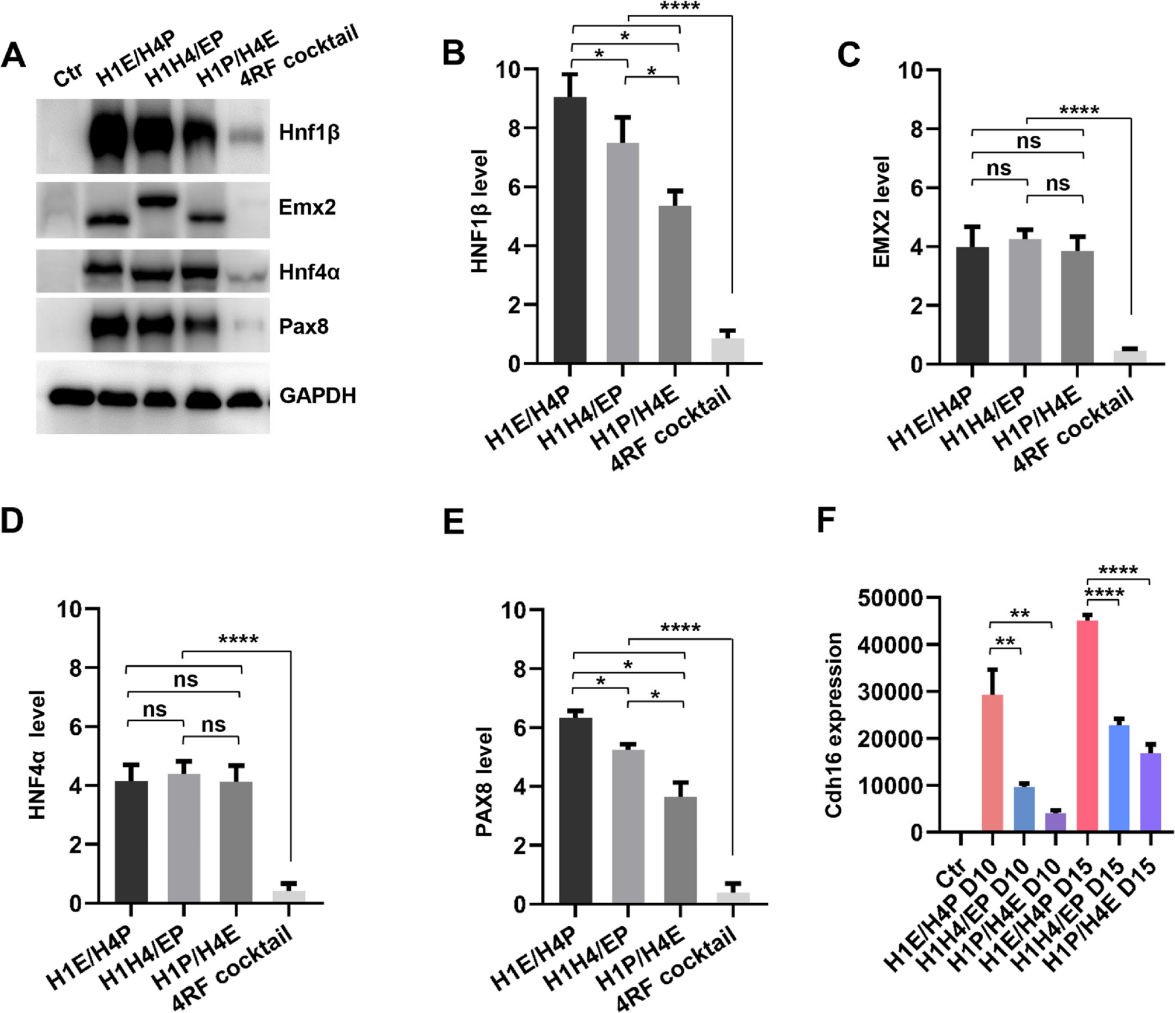
Disparate RF combinations result in different expression levels of HNF1β, EMX2, PAX8 and HNF4α. **A** Western blots analyses of H1, E, H4, and P protein levels in MEFs infected by H1E/H4P, H1H4/EP, H1P/H4E, and 4RF cocktail at 5 days. GAPDH was used as a loading control. **B-E** Quantification analyses of HNF1β, EMX2, HNF4α, and PAX8 proteins. (H1E/H4P, H1H4/EP, H1P/H4E) vs 4RF cocktail using one-way ANOVA with Tukey multiple comparisons test. H1E/H4P vs H1H4/EP, H1E/H4P vs H1P/H4E, H1H4/EP vs H1P/H4E using Student’s *t*-test. **F** RT-qPCR analyses of renal epithelial Cdh16 in MEFs infected with H1E/H4P, H1H4/EP, and H1P/H4E combinations compared to Ctr after 10 and 15 days of culture, *n* = 3 samples. Quantifications (**B**, **C**, **D**, **E**, and **F**) are represented as mean ± SEM, *P< 0.05, **P < 0.01, ****P < 0.001, ns, not significant. Student’s *t*-test, one-way ANOVA with Tukey multiple comparisons test

Different sets of bicistronic RF cassettes resulted in varying reprogramming efficiencies, possibly due to distinct levels and ratios of H1, E, P, and H4 protein expression. We performed western blot (WB) analyses to assess the expression levels and ratios of H1, E, P, and H4 in MEFs infected with 3 sets of bicistronic RF cassettes and 4RF cocktail (Fig. 2A). WB detected H1, E, P, and H4 proteins at the appropriate molecular weight (Fig. S2D**-**G). We noticed that EMX2 in H1H4/EP combination, HNF4α in H1E/H4P and H1P/H4E combinations displayed a noticeable molecular weight shift (Fig. 2A), which could be due to linkage of P2A to EMX2 and HNF4α in these cassettes (Fig. S2H). After normalization to the loading control, quantification of band intensities revealed that each set of dual bicistronic combinations produced distinct expression levels of H1, E, P, and H4. We found that transduction of bicistronic RF combinations generated much high levels of HNF1β, EMX2, HNF4α and PAX8 proteins (Fig. 2A-E). In contrast, infection of 4RF cocktail produced heterogenous and very low levels of four RFs (Fig. 2A), which was probably due to random infections of separate RFs without selection. Among the bicistronic RF cassettes, H1E/H4P and H1H4/EP combinations expressed relatively high levels of HNF1β and PAX8 with a comparable and low level of EMX2 and HNF4α, a distinct expression ratio or stoichiometry, which was determined by quantification of band intensities in Western blots (Fig. 2B-E). We found that both HNF1β and PAX8 protein levels were higher in H1E/H4P combination than H1H4/EP. Furthermore, H1P/H4E cassette expressed the lowest levels of HNF1β and PAX8 among the three bicistronic cassettes. Given that H1E/H4P combination resulted in the most efficient iREC induction compared with 4RF cocktail and other bicistronic cassettes, we conclude that the efficient induction of iREC reprogramming is facilitated by maintaining the high expression levels of RFs with balanced stoichiometry (i.e., higher expression levels of H1 and P8 with lower levels of E2 and H4), achieved in the H1E/H4P combination. As a result, the endogenous renal epithelial marker *Cdh16* was induced substantially high by H1E/H4P transduction at day 10 and day 15 of culture (Fig. 2F). We also tested the capacity of HE/H4P cassette on converting human fibroblasts to iRECs. Transfections of human fibroblastic cells with H1E/H4P, including human normal diploid fibroblasts (IMR90) and human skin fibroblasts (HSF), resulted in a significant increase in renal transcriptional factors and epithelial cell markers, as well as a reduction in fibroblast specific genes (Fig. S2I**-**L), which holds the potential for reprogramming human primary fibroblasts into renal epithelial cells.

### Improving the efficiency and quality of iREC induction by H1E/H4P combination

**Fig. 3:**
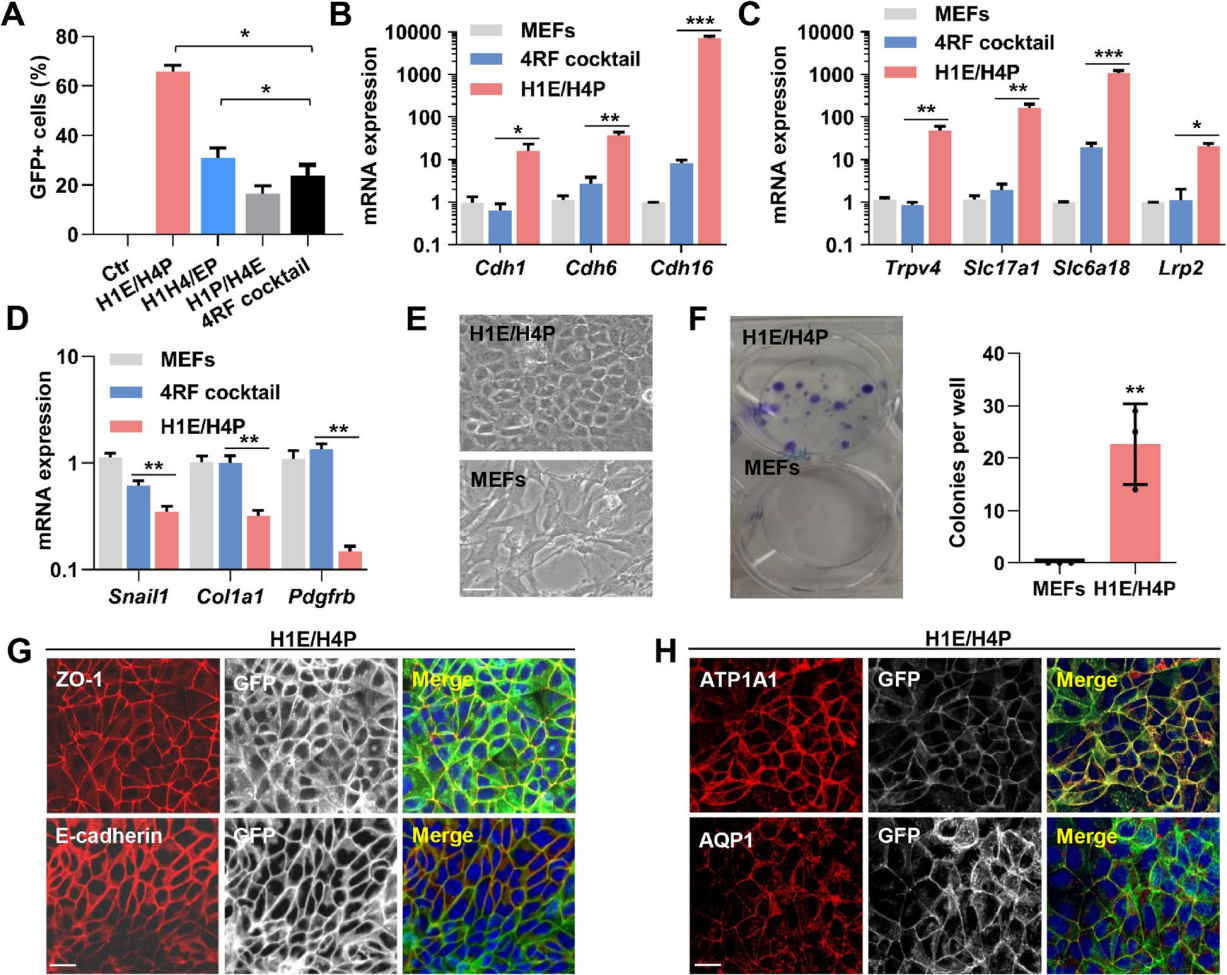
Enhancement of iRECs reprogramming efficiency and quality by H1E/H4P Combination. **A** Bar charts depicting percentage of GFP^+^ iRECs in H1E/H4P, H1H4/EP, H1P/H4E combinations, and 4RF cocktail at 5 weeks of transduction by FASC analyses, *n* = 3 samples. **B**, **C** Relative mRNA expression levels of cadherin superfamily genes (**B**), renal ion transporters and *Lrp2* (**C**) after 9 days with H1E/H4P, 4RF cocktail, and lacZα transduced MEFs, as determined by qPCR. *n *= 3 samples. **D** RT-qPCR analysis of fibroblastic and EMT markers in MEFs transduced with H1E/H4P, 4RF cocktail, and lacZα, *n *= 3 samples. **E** Phase-contrast images of H1E/H4P induced iRECs and MEFs showing epithelial and mesenchymal cell morphologies, respectively. **F** Colony formation of iRECs and MEFs assessed by crystal violet staining, and quantification of colonies formed by MEFs and iRECs after 2 weeks of culture, *n *= 3 wells. **G** Immunofluorescence staining of the indicated epithelial marker proteins in GFP^+^ iRECs with anti-E-cadherin (red), and anti-ZO-1 (red) antibodies. Nuclei were stained with DAPI. **H** Confocal images of GFP^+^iRECs with renal epithelial expression proteins with anti-ATP1A1 (red), and anti-AQP1 (red) antibodies. Quantifications (**A**, **B**, **C**, **D**, and **F**) are represented as mean ± SEM, *P < 0.05, **P < 0.01, ***P < 0.001. Student’s *t*-test. Scale bars, 20 µm (**D**, **G**, **H**)

We next focused on the improvement of reprogramming efficiency and biological properties of iRECs induced by H1E/H4P cassette. We found that transduction of H1E/H4P substantially increased the percentage of Cdh16-mGFP^+^ cells to 65.7% after puromycin and blasticidin S selection for five weeks (Fig. 3A), much higher than separate H1, E, P, and H4 transduction (23.8%) (Kaminski et al. 2016). RT-qPCR detected the substantial upregulation of cadherin superfamily genes (*Cdh1*, *Cdh6*, and *Cdh16)*, ion and amino acid transporter genes (*Trpv4*, *Slc17a1*, and *Slc6a18)*, apolipoprotein-receptor megalin (*Lrp2*) (Fig. 3B, C) and renal transcriptional factors (*Hnf1α*, *Lhx1*, and *Pax2*) (Fig. S3A) in H1E/H4P-transduced MEFs, in comparison with MEFs with transduction of H1, E, P, H4 cocktail. By contrast, a significant decline in the expression of fibrosis and EMT-related genes (*Snail1*, *Col1a1*, and *Pdgfrb*) was observed in H1E/H4P-transduced MEFs compared with 4RF cocktail transduction (Fig. 3D). Thus, H1E/H4P combination with antibiotics selection affords a more efficient conversion of MEFs into renal epithelial cell fates. We next subjected H1E/H4P-induced iRECs to morphological and immunostaining analyses. These iRECs exhibited the characteristic cobblestone-like appearance of epithelial cells (Fig. 3E), and formed colonies when sparsely seed, in comparison with MEFs (Fig. 3F). Immunostaining analyses revealed the membrane localization of epithelial cell marker ZO-1, E-cadherin, and Epcam in iRECs (Fig. 3G, S3B), but not in MEFs (Fig. S3C-E). Notably, the renal tubular markers Na^+^/K^+^-ATPase (ATP1A1) and aquaporin 1 (AQP1) were detectable at the iREC membrane (Fig. 3H) (He et al. 2018; Ware et al. 2017). In contrast, Vimentin marking mesenchymal cells, was absent in iRECs but present in MEFs (Fig. S3F, G). These findings indicate that H1E/H4P-induced iRECs acquired epithelial cell characters by loosing mesenchymal properties. We also analyzed the expression of renal epithelial cell markers in 4RF cocktail-induced iRECs, and observed the limited expression of ZO-1, E-cadherin and Epcam (Fig. S3H), compared with H1E/H4P transduction (Fig. 3G, S3B). In addition, only a minority of GFP^+^ iRECs express renal tubular cell markers ATP1A1 and AQP1 (Fig. S3I), confirming the superiority of H1E/H4P cassette in enhancing the iREC quality.

**Fig. 4:**
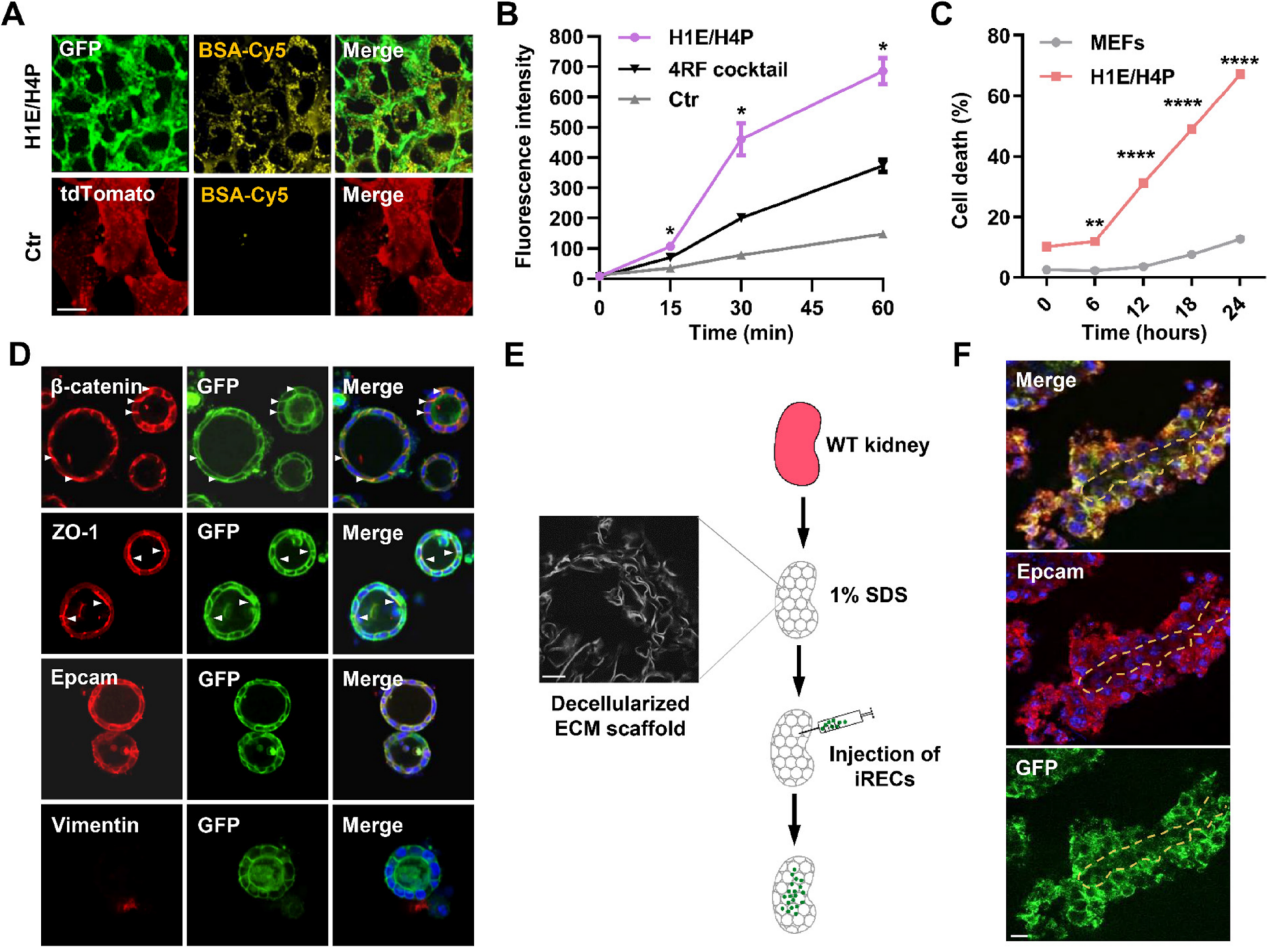
H1E/H4P-induced iRECs display functional characteristics of renal tubular epithelial cells. **A** Representative images of Ctr and H1E/H4P induced iRECs incubated with Cy5-labelled albumin. **B** Quantification of albumin uptake in Ctr, 4RF cocktail and H1E/H4P induced iRECs over a 60 min time course, *n* = 3 visual fields per time point. **C** Percentage of dead cells in cisplatin (6 µg/ml)-treated MEFs and iRECs over 24 h time course, *n* = 3 wells per time point. **D** Confocal images showing the indicated proteins in iRECs grown in 3D Matrigel, stained with anti-β-catenin (red), anti-ZO-1 (red), anti-Epcam (red), and anti-Vimentin (red) antibodies. Nuclei were stained with DAPI. Arrowheads indicate basolateral localization of β-catenin and apical localization of ZO-1. **E** The schematic diagram illustration of the decellularized kidney scaffold construction and cell implantation assay (**E**). Wild-type (WT) kidneys were decellularized and injected with iRECs. **F** Confocal images of decellularized kidneys repopulated with H1E/H4P induced iRECs stained with anti-Epcam (red) and anti-GFP antibodies. Nuclei were stained with Hoechst; ECM, extracellular matrix; Quantifications (**B**, and **C**) are represented as mean ± SEM, *P < 0.05, ****P < 0.0001, Student’s *t*-test. Scale bars, 15 µm (**A**), 50 µm (**D**), 30 µm (**E**), 10 µm (**F**)

Given that the expression of *Lrp2*, an endocytic receptor mediating the endocytotic uptake of proteins (Cui et al. 1996), is highly upregulated in H1E/H4P-induced iRECs (Fig. 3C), we assessed the uptake of fluorescently labelled albumin (BSA-Cy5) and observed significantly increased Cy5 fluorescence intensity in iRECs (Fig. 4A). By contrast, MEFs failed to arrogate BSA-Cy5. Furthermore, H1E/H4P-induced iRECs manifested the enhanced uptake dynamics of BSA-Cy5 in comparison with MEFs transduced with 4RF cocktail or retroviral backbone (Fig. 4B) (Kaminski et al. 2016). We next exposed iRECs to cisplatin, a nephrotoxicant that causes acute apoptosis of tubular cells in the kidney (Kong et al. 2018), and observed apoptotic cells as early as 6 h in iRECs, whereas cisplatin-treated MEFs grew sustainably (Fig. 4C, S4A), suggesting that iRECs respond to nephrotoxic substance as renal tubular cells. To assess whether iRECs develop morphological tubule-like structures, we cultured iRECs in 3-dimensional Matrigel matrix (Fig. S4B). When grown in a Matrigel matrix, iRECs formed spherical structures with a central lumen (Fig. 4D). The spheroids derived from iRECs exhibited an apical-basal polarity, as evidenced by β-catenin basolateral localization and ZO-1 apical localization. We also observed the intercellular interfaces accumulation of Epcam in iREC spheres, and an absence of mesenchymal marker Vimentin (Fig. 4D). Lastly, we assessed if iRECs can assemble into tubules in decellularized scaffolds, which were guided only by extracellular matrix. Kidneys were harvested from mouse donors and stripped of cellular materials as decellularized scaffolds. We injected iRECs into the scaffold and observed that iRECs grew and organized into partially convoluted tubules (Fig. 4E, F), in which Epcam marks the continuous basement membrane between neighboring iRECs (Fig. 4F). Altogether, these findings demonstrate that H1E/H4P retroviral cassette improves the efficiency and quality of iREC reprogramming.

## Discussion

Since the initial study demonstrating that the exogenous expression of four renal RFs can directly convert MEFs into functional iRECs (Kaminski et al. 2016), several studies have been undertaken to improve the reprogramming efficiency of iRECs, albeit with a focus on iRECs associated with clinical mutations of renal disorders (Grand et al. 2023; Marchesin et al. 2019). However, significant advancements in enhancing the reprogramming efficiency and quality of iRECs remain elusive. In this study, we have developed an advanced bicistronic vector system with antibiotics selection that ensures each fibroblast expresses four reprogramming factors, thus improving iREC induction. Furthermore, we find that the high and balanced expression of RFs contributes to the improved reprogramming efficiency. Among the retroviral bicistronic cassettes, we reveal that H1E/H4P cassette substantially improves iREC induction and biological quality. The H1E/H4P-induced iRECs exhibit the superior attributes of renal epithelial cells, including expressing renal tubular-specific genes, acquiring epithelial cell characters, and forming spherical structures with a polarity.

Previous approach induces iRECs formation with transduction of pooled viruses encoding 4 individual RFs (Kaminski et al. 2016).This generates uncontrollable and heterogeneous expression of RFs in fibroblasts, resulting in low and variable reprogramming efficiencies. Polycistronic viral system has been successfully utilized in various cell reprogramming through balancing reprogramming factor expression, limiting viral insertions and enhancing transdifferentiation efficiency. For instance, in iCM induction, *Mef2c* (M)-*Gata4* (G)-*Tbx5* (T) polycistronic retroviral transduction generates optimal stoichiometry of M, G, T and results in a prominent increase in efficiency and quality of iCM reprogramming (Wang et al. 2015; Ma et al. 2015). Similarly, a polycistronic cassette encoding *OCT4* (O), *NANOG* (N), *SOX2* (S) and *LIN28* (L) on a single retroviral vector achieves the most efficient system to induce pluripotent stem cells while limiting genomic integrations (Jung et al. 2014). In this study, we have developed bicistronic retroviral system for iREC reprogramming that offers numerous advances over existing approaches. First, PuroR and BSD are tagged to retroviral vectors to ensure the selection of MEFs containing 4 RFs, thus improving iREC induction. Second, the desired stoichiometry of RFs achieved in bicistronic H1E/H4P cassette further contributes to the improved efficiency and quality of iRECs. Third, MEFs harboring Cdh16-Cre; mT/mG mice allele are utilized for iREC reprogramming analysis, enabling directly monitoring single cell fate conversion. We reason that, during iREC direct reprogramming, the four RFs act in concert at regulatory elements to rapidly silence fibroblast gene expression and synergistically activate the expression of renal epithelial genes. The high expression levels and desired stoichiometry of H1, E, H4 and P either facilitates or limits one another’s ability to bind to a large amount of gene regulatory elements, correlating with opening or closure of chromatin structure at particular loci. In addition, many renal gene regulatory elements could be sequentially activated through cooperative interactions with reprogramming factors to further augment renal epithelial cell attributes. Thus, the high and balanced expression of H1, E, H4, P proteins are required to orchestrate epigenetic and transcriptional landscape that governs iREC reprogramming.

It has been reported that fibroblasts can be in vivo reprogrammed to cardiomyocytes, oligodendrocytes, and hepatocytes (Qian et al. 2012; Rezvani et al. 2016; Song et al. 2016; Wang et al. 2020a). However, there are no studies to date with regard to iREC reprogramming in vivo. Future studies will be focused on investigation of in vivo conversion of fibroblasts to iRECs upon kidney injury. It will be also important to identify a minimal set of RFs that can be placed on a single viral vector for iREC induction in the context of kidney injury and human disease. Overall, our study has developed a bicistronic retroviral system that significantly enhances reprogramming efficiency and biological quality of iRECs, a crucial step towards potential applications in renal injury repair and regeneration.

## Materials and Methods

### Animals

*Gt(ROSA)26Sor*^*tm4(ACTB-tdTomato, -EGFP) Luo/J*^ mice were purchased from The Jackson Laboratory (no.007676, strain of origin: B6.129(Cg)) and bred with Ksp-Cre/+(B6.Cg-Tg(Cdh16-cre)91Igr/J, #012237) mice. All mice used were on a C57BL/6J genetic background. Mice were housed at controlled temperature (25 °C) and 12-hour light/12-hour dark cycle. Breeding and genotyping were performed according to standard procedures. All animal experiments conformed to the regulations drafted by the Association for Assessment and Accreditation of Laboratory Animal Care in Shanghai and were approved by the East China Normal University Center for Animal Research (no. m20210806).

### Cell culture

To generate MEFs carrying the reporter system, we crossed the mTmG homozygotes with Cdh16-Cre/+ and isolated embryos at E12.5 to E14.5. Embryos were rinsed in PBS, and the head, tail, and all innards were removed. The remaining tissue was chopped and incubated with 0.05% trypsin/EDTA (Gibco, 25200072) for 15 min at 37 °C. Trypsinization was stopped by addition of DMEM containing 10% FBS, then cells from one embryo were plated into a 25 cm^2^ culture flask. MEFs were cultured in DMEM supplemented with 10% FBS and 1% penicillin/streptomycin (Gibco,15140122). Plat-E packaging cells were maintained in growth media containing DMEM plus 10% FBS, 0.1 mM non-essential amino acids, 2 mM GlutaMAX, 1% penicillin/streptomycin, 1 µg/ml of puromycin (Selleck, S7417), and 10 µg/ml of blasticidin S (Selleck, S7419).

### Bicistronic plasmid construction

Full-length cDNAs of H1, E, H4, and P genes were amplified by PCR from mouse inner medullary collecting duct cells (IMCD3) and cloned into pMXspuro-GFP (Addgene: 74203) for retroviral packaging. To generate the pMXs-BSD vector conferring blasticidin S resistance, the pMXs vector was digested with Hind III / SGRDI to remove the *PuroR* gene. Subsequently, the *BSD* gene was amplified from LentiV_Cas9_Blast (Addgene: 125592) and inserted into the pMXs vector. Bicistronic retroviral units, including H1-P2A-E (H1E), H4-P2A-P (H4P), H1-P2A-H4 (H1H4), E-P2A-P (EP), H1-P2A-P (H1P), and H4-P2A-E (H4E) were PCR amplified and sequentially cloned into the pMXs vectors. To construct pMXs-lacZα mock retroviral vectors, lacZα was PCR and subcloned into a pMXs vector. Expression vectors were generated via ClonExpress™ MultiS one-step cloning kit (Vazyme, C113).

### Retrovirus generation and infection

To produce retroviruses, pMXs-lacZα, H1-P2A-E (H1E), H4-P2A-P (H4P), H1-P2A-H4 (H1H4), E-P2A-P (EP), H1-P2A-P (H1P), and H4-P2A-E (H4E) bicistronic retroviral plasmids were transfected into Plat-E cells as previously described (Wang et al. 2020b). We introduced pMXs-based retroviral vectors into Plat-E cells using Lipofiter Transfection Reagent (Hanbio, HB-LF). At 48 h post-transfection, we collected the virus-containing supernatant, filtered it through 0.45 μm filter, and concentrated it with PEG8000 (Sigma, P5413) overnight. We resuspended viruses in DMEM supplemented with 4 µg/ml polybrene (40804ES76, Yeasen Biotech) and added them to MEFs immediately. Twenty-four hours after infection, the medium containing the virus was replaced with DMEM media. The medium was then replaced every 2–3 days thereafter. To perform positive selection on MEFs, MEF media supplemented with puromycin at 2 µg/ml and blasticidin S at 20 µg/ml for three days.

### Retrovirus concentration and titer

Retroviral titration was conducted using the Retro-X qRT-PCR Titration Kit (Clontech) according to the manufacturer’s instructions. pMXs retroviral vectors encoding cDNAs of H1-P2A-E (H1E), H4-P2A-P (H4P), H1-P2A-H4 (H1H4), E-P2A-P (EP), H1-P2A-P (H1P), H4-P2A-E (H4E) were transfected into Plat-E cells. At 48 h post-transfection, the virus-containing supernatant was harvested. Viral RNAs were extracted from an aliquot of the supernatant. Serial dilutions of the viral RNA were subjected to qRT-PCR to determine the threshold cycle (Ct) values for each dilution. The RNA genome copy number in each dilution was then calculated by correlating the Ct values with a standard curve. Concentrated retroviruses were serially diluted and infected 4 × 10^4^ MEFs, and the number of transduced cells was determined by FACS analyses. Retroviral titer, defined as p.f.u per ml, was calculated by multiplying the number of infected cells by dilution factor and 1/V, where V is the volume of virus.

### Calculating the Multiplicity of Infection (MOI)

To determine the optimal multiplicity of infection (MOI), we performed a titration of the retrovirus on MEFs using incremental volumes ranging from 1 µL to 120 µL. This titration aimed to identify the virus concentration that achieves a high infection rate without compromising cell viability. Viral infection efficiency was assessed using flow cytometry, providing quantitative measure of the proportion of infected cells. The multiplicity of infection (MOI) was calculated as the ratio of the number of viral particles to the number of target cells.

### Induce human fibroblast to renal epithelial cell transdifferentiation

pCDH-CMV (Addgene: 72265) lentiviral vectors carrying HNF1β-P2A-EMX2 and HNF4α-P2A-PAX8 were introduced into 293T cells using Lipofiter Transfection Reagent (Hanbio, HB-LF), along with the packaging plasmid psPAX2 (Addgene: 12260) and the envelope plasmid pMD2.G (Addgene: 12559), to produce viruses. Human normal diploid fibroblasts (IMR90) and human skin fibroblasts (HSF) were infected with H1E/H4P viruses in the presence of 8 µg/ml polybrene. Twelve hours post-infection, the medium was replaced with fresh DMEM. For positive selection of human fibroblasts, the medium was supplemented with 1 µg/ml puromycin and 10 µg/ml blasticidin S for three days. Twelve days after viral transduction, cells were harvested for RNA extraction and subsequent qRT-PCR analysis to detect the expression of renal tubular epithelial cell marker genes and fibroblast marker genes.

### Western blot analysis

For Western blot analysis, cells were collected and homogenized in the RIPA buffer (NCM Biotech, WB3100) supplemented with protease inhibitor cocktail (APExBIO, K1008). The protein concentrations of lysates were determined using the BCA method (Thermo Scientific, 23227). Samples were mixed with the 5× loading buffer (Epizyme Biotechnology, LT103) and incubated for 10 min at 98 °C. 20–30 µg of total proteins were resolved by SDS-PAGE and transferred to PVDF membranes (Millipore, IPVH00010). Immunoblots were blocked with 5% non-fat milk in Tris-buffered saline (TBS), and probed with indicated primary antibodies overnight at 4 °C. After washing in TBST three times, blots were incubated with horseradish peroxidase-conjugated secondary antibodies (1:5000) diluted in TBST supplemented with 3% BSA for 1 h at room temperature. Immunoreactivity was developed with enzymatic detection using LumiBest enhanced chemiluminescence (Shanghai Share-bio biotechnology, SB-WB011). Antibodies used for western blot in this study were as follows: Anti-HNF1B (Abcam, ab128912, 1:1000), Anti-EMX2 (Sigma, HPA065294, 1:1000), Anti-HNF4A (Abcam, ab181604, 1:1000), Anti-PAX8 (Abcam, ab239363, 1:1000), Anti-GAPDH (Abcam, ab8245, 1:5000), Anti-Peptide 2 A Antibody (Sigma, MABS2005,1:1000), HRP-anti-rabbit IgG (Cwbio, CW0103, 1:5000) and HRP-anti-mouse IgG (Cwbio, CW0102S, 1:5000). In order to quantify HNF1β, EMX2, HNF4A, and PAX8, three parameters were taken into account in our experiments. First, the amounts of uploaded proteins were adjusted to be equal and avoid over loading. Second, the concentrations of antibodies were calibrated to prevent nonlinear saturation. Third, the chemiluminescent HRP substrates exhibited linear relationships in the quantitative application. Protein levels were determined by densitometry-based quantification, in which band intensities in the immunoblots were measured and analyzed using ImageJ software. Signal intensities were normalized to the loading control GAPDH signal.

### Immunofluorescent staining

For Immunofluorescent staining, cells were washed with PBS twice and then fixed with 4% paraformaldehyde (PFA) (Sigma, 158127) for 15 min at room temperature. For permeabilization, cells were then incubated in 0.1% Triton X-100 (Sangon Biotech, A600198) in PBS for 15 min at room temperature. After being washed three times with PBS, cells were then blocked in a solution of PBS containing 5% BSA for 60 min at 37 °C. Cells were incubated with primary antibodies overnight at 4 °C next with a secondary fluorescence conjugated antibody for 60 min at 37 °C in the dark. Primary and secondary antibodies were diluted in a solution of PBS containing 1% BSA and 0.1% Triton X-100. Primary antibodies used in this study were as follows: Anti-Epcam (Abcam, ab71916, 1:200), Anti-ZO-1 (Abcam, ab221547, 1:200), Anti-E-cadherin (Abcam, ab1416, 1:200), Anti-ATP1A1 (Thermo Scientific, A-11120, 1:250), Anti-AQP1 (Santa Cruz, sc-515770, 1:200) and Anti-Vimentin (Thermo Scientific, ab8978, 1:200). Secondary antibodies used in this study were as follows: goat anti-rabbit 488 (Thermo Scientific, A-11008, 1:800), goat anti-rabbit 594 (Thermo Scientific, A-11012, 1:800), goat anti-mouse 488 (Thermo Scientific, A-11001, 1:800), and goat anti-mouse 594 (Thermo Scientific, A-11005, 1:800). Images were taken by Dragonfly High-Speed Confocal Microscope System (Andor). Image analysis was performed using Fusion (Andor) and Imaris (Bitplane) software.

### 3D cell culture

Cells were trypsinized and then passed through a 40 μm cell strainer before counting. Cells were resuspended 1:1 in 100 µl growth factor-reduced Matrigel (Corning, 354230) and seeded onto Lab-Tek™ II chamber slides (Thermo Scientific, 155360). Renal epithelial growth medium (Lonza, CC-3190) was added after Matrigel was polymerized for 15 min at 37 °C. Spheroid formation was evaluated qualitatively after 7 days and photographically documented. For immunofluorescence staining, cells were fixed in 4% PFA for 30 min at room temperature. Cells were then incubated at 4 °C for 30 min.

### Albumin uptake assay

Cells were incubated with Cy5-labelled albumin (Ruixibio, R-FB-006) at 37 °C for 0, 15, 30, and 60 min. After incubation, Images were taken by Dragonfly High-Speed Confocal Microscope Systems (Andor). Image analysis was performed using Fusion (Andor) and Imaris (Bitplane) software.

### FACS analyses and sorting

For flow cytometry analyses, cells were digested with 0.05% trypsin, resuspended in FACS buffer (PBS supplemented with 3% FBS and 5 mM EDTA), filtered through a 40 μm cell strainer and analyzed using the LCR Fortessa FACS analyzer (Becton Dickinson). For the sorting of GFP^+^ cells, the ARIA III cell sorters (Becton Dickinson) were used. Data were analyzed by FlowJo (Becton Dickinson) software.

### Quantitative real-time PCR

Total RNAs were extracted using the QIAGEN RNeasy Mini Kit (QIAGEN, 74104). RNA (1 µg) was reversely transcribed using PrimeScript II 1st Strand cDNA Synthesis Kit (TaKaRa, 6210 A). qPCR was carried out on biological triplicates with SYBR Premix Ex Taq II (TaKaRa, RR041A) using a Roche LightCycler 480 II system. The relative expression levels of target genes were normalized to β-actin and quantified by the 2^−ΔΔCT^ method. Primers are documented in the Table S2.

### Cisplatin assay

MEFs and iRECs were incubated with 6 µg/ml cisplatin (T1564, Teva). Cells were harvested by collecting both the supernatants and the attached cells. Drug-treated cells were stained with 1 µg/ml of DAPI (D1306, Invitrogen) for 5 min and fluorescence was detected by flow cytometry.

### Decellularized renal scaffold construction and cell implantation

For recellularization experiments, the kidneys of adult wild-type mice were rinsed 10 times with heparinized PBS 10 U/ml (Sangon Biotech, A603251). Then, they were incubated in 1% SDS (Sangon Biotech, A600485) in PBS for a period of three to five days until decellularization was observable, followed by 1% Triton X-100 for 24 h. Subsequently, the decellularized kidneys were flushed with PBS containing 1% penicillin/streptomycin and 100 µg/ml Primocin (Invivogen, ant-pm-1). After γ sterilization with an irradiation dose of 3 kGy, 10^6^ iRECs were seeded into the organ scaffolds by injection using a 25G needle. The cultivated cells were then incubated in DMEM plus 10% FBS for a duration of 14 days. The organ scaffolds were fixed in 4% PFA at 4 °C overnight and following histological experiments were performed using 50 μm cryosections.

### Statistical analysis

GraphPad software was used to perform statistical analysis. Data were analyzed by the two-tailed Student’s *t*-test and one-way ANOVA with Tukey multiple comparisons test, data are represented as mean ± SD or mean ± SEM. and considered significant at *P* < 0.05.

## Supplementary Information

Supplementary Material 1: Fig S1. Analyses of retroviruses titer and MOI. (A) The titers of bicistronic RF retroviruses including H1-P2A-E (H1E), H4-P2A-P (H4P), H1-P2A-H4 (H1H4), E-P2A-P (EP), H1-P2A-P (H1P), and H4-P2A-E (H4E). The titers were calculated based on FACS analysis of MEFs infected by a series of retrovirus dilutions (see Methods). (B) FACS analysis of the MEFs infected with retroviruses. Multiplicity of Infection (MOI) was calculated and shown in each condition (see Methods). Fig S2. Characterizations of iRECs reprogramming efficiencies and gene expression levels. (A, B) FACS analyses for the percentage of GFP^+^ cells at 2 and 3 weeks of reprogramming in H1E/H4P, H1H4/EP, and H1P/H4E. (C) qPCR analyses of endogenous *Hnf1β*, *Emx2*, *Hnf4α*, and *Pax8* induced in MEFs transduced by H1E/H4P, H1H4/EP, and H1P/H4E in 3 weeks, compared to Ctr. PCR primers were designed to located in the 3’ UTR of targeted mRNA of each gene. *n*=3 samples. (D-G) Immunoblotting showing H1, E, H4, or P protein on transduced MEFs at 3 days. (H) Western blot analyses detecting EMX2 shift bands in H1H4/EP and HNF4α shift bands in H1E/H4P, H1P/H4E. (I, J) Expression of renal epithelial genes *HNF1A*, *CDH16*, *EPCAM*, *GGT1* and fibroblast gene *COL1A1*, *COL3A1* were examined using qPCR analyses in H1E/H4P infected IMR90 cells. *n*=3 samples. (K, L) qPCR analyses of renal epithelial genes *HNF1A*, *CDH16*, *EPCAM*, *GGT1* and fibroblast gene *COL1A2*, *MMP14* in H1E/H4P treated HSF cells. *n*=3 samples. Quantifications (C, I-L) are represented as mean ± SEM, *P < 0.05, ***P < 0.001, ****P < 0.0001, Student’s *t*-test. Fig S3. Characterizations of iRECs and MEFs. (A) Relative mRNA expression levels of renal transcription factors *Hnf1α*, *Lhx1*, and *Pax2* as determined by RT-qPCR in control and H1E/H4P infected MEFs, *n* = 3 samples. (B) Immunostaining of H1E/H4P-induced iRECs for the epithelial protein Epcam. (C-E) Immunofluorescence staining of the epithelial makers ZO-1, Epcam, and E-cadherin in MEFs. (F, G) Immunostaining of mesenchymal protein Vimentin in H1E/H4P-induced iRECs and MEFs. Nuclei were stained with DAPI. (H) Immunofluorescent images of epithelial makers ZO-1, Epcam, and E-cadherin in 4RF cocktail-induced iRECs. (I) Immunofluorescent images of renal epithelial marker ATP1A1 and AQP1 in 4RF cocktail-induced iRECs. Quantifications (A) are represented as mean ± SEM, *P < 0.05, ***P < 0.001, ****P < 0.0001, Student’s *t*-test. Scale bars, 20 µm (B-I). Fig S4. H1E/H4P-induced iRECs show properties of renal tubular epithelial cells. (A) Percentage of dead cells (%) in cisplatin (6 µg/ml)-treated MEFs and iRECs, *n*=3 samples. (B) A diagram exhibiting the timeframe of 3D cell culture for iRECs.

Supplementary Material 2: Table S1: Raw data for the percentage of GFP^+^ cells at 2 weeks and 3 weeks of reprogramming in H1E/H4P, H1H4/EP, and H1P/H4E.

Supplementary Material 3: Table S2: Primer sequences for RT-qPCR.

## Data Availability

The data used to support the findings of this study are included within the article.

## Abbreviations

iRECs: Induced renal epithelial cells
RFs: Reprogramming factors
H1: Hnf1β
E: Emx2
P: Pax8
H4: Hnf4α
MEFs: Mouse embryonic fibroblasts
iCM: Induced cardiomyocytes
G: Gata4
M: Mef2c
T: Tbx5
PuroR: Puro Resistance gene puromycin N-acetyltransferase
BSD: Blasticidin S deaminase
MOI: Multiplicity of infection
FACS: Fluorescence-activated cell sorting
WB: Western blot
