# Correction: Expression levels and stoichiometry of Hnf1β, Emx2, Pax8 and Hnf4α influence direct reprogramming of induced renal tubular epithelial cells

**DOI:** 10.1186/s13619-024-00210-0

**Published:** 2024-12-02

**Authors:** Xueli Hu, Jianjian Sun, Meng Wan, Bianhong Zhang, Linhui Wang, Tao P. Zhong

**Affiliations:** 1 Shanghai Key Laboratory of Regulatory Biology, Institute of Molecular Medicine, School of Life Sciences, East China Normal University, Shanghai, 200241 China; 2 Guangdong Cardiovascular Institute, Guangdong Provincial People’s Hospital, Guangdong Academy of Medical Sciences, Guangzhou, Guangdong 510100 China; 3 Department of Urology, Changhai Hospital, Shanghai, 200433 China

**Correction to: Cell Regen 13, 19 (2024)**

**https://doi.org/10.1186/s13619-024-00202-0**

Following publication of the original article (Hu et al. 2024), the authors reported an error in the article title, it needed to be changed from “Expression levels and stoichiometry of Hnf1β, Emx2, Pax8 and Hnf4 influence direct reprogramming of induced renal tubular epithelial cells” to “Expression levels and stoichiometry of Hnf1β, Emx2, Pax8 and Hnf4α influence direct reprogramming of induced renal tubular epithelial cells”.

The correct article title has been provided in this Correction.

The original article (Hu et al. 2024) has been corrected.
