# Supplementary material for: Expression levels and stoichiometry of Hnf1β, Emx2, Pax8 and Hnf4α influence direct reprogramming of induced renal tubular epithelial cells
Source: Cell Regen. 2024 Sep 30;13:19. doi: 10.1186/s13619-024-00202-0 (PMC11442758; doi:10.1186/s13619-024-00202-0)
Supplement: Supplementary file 1 — Supplementary Material 1: Fig S1. Analyses of retroviruses titer and MOI. (A) The titers of bicistronic RF retroviruses including H1-P2A-E (H1E), H4-P2A-P (H4P), H1-P2A-H4 (H1H4), E-P2A-P (EP), H1-P2A-P (H1P), and H4-P2A-E (H4E). The titers were calculated based on FACS analysis of MEFs infected by a series of retrovirus dilutions (see Methods). (B) FACS analysis of the MEFs infected with retroviruses. Multiplicity of Infection (MOI) was calculated and shown in each condition (see Methods). Fig S2. Characterizations of iRECs reprogramming efficiencies and gene expression levels. (A, B) FACS analyses for the percentage of GFP+ cells at 2 and 3 weeks of reprogramming in H1E/H4P, H1H4/EP, and H1P/H4E. (C) qPCR analyses of endogenous Hnf1β, Emx2, Hnf4α, and Pax8 induced in MEFs transduced by H1E/H4P, H1H4/EP, and H1P/H4E in 3 weeks, compared to Ctr. PCR primers were designed to located in the 3’ UTR of targeted mRNA of each gene. n=3 samples. (D-G) Immunoblotting showing H1, E, H4, or P protein on transduced MEFs at 3 days. (H) Western blot analyses detecting EMX2 shift bands in H1H4/EP and HNF4α shift bands in H1E/H4P, H1P/H4E. (I, J) Expression of renal epithelial genes HNF1A, CDH16, EPCAM, GGT1 and fibroblast gene COL1A1, COL3A1 were examined using qPCR analyses in H1E/H4P infected IMR90 cells. n=3 samples. (K, L) qPCR analyses of renal epithelial genes HNF1A, CDH16, EPCAM, GGT1 and fibroblast gene COL1A2, MMP14 in H1E/H4P treated HSF cells. n=3 samples. Quantifications (C, I-L) are represented as mean ± SEM, *P < 0.05, ***P < 0.001, ****P < 0.0001, Student’s t-test. Fig S3. Characterizations of iRECs and MEFs. (A) Relative mRNA expression levels of renal transcription factors Hnf1α, Lhx1, and Pax2 as determined by RT-qPCR in control and H1E/H4P infected MEFs, n = 3 samples. (B) Immunostaining of H1E/H4P-induced iRECs for the epithelial protein Epcam. (C-E) Immunofluorescence staining of the epithelial makers ZO-1, Epcam, and E-cadherin in MEFs. (F, G) Immunostaining [file 13619_2024_202_MOESM1_ESM.pptx]

## Slide 1
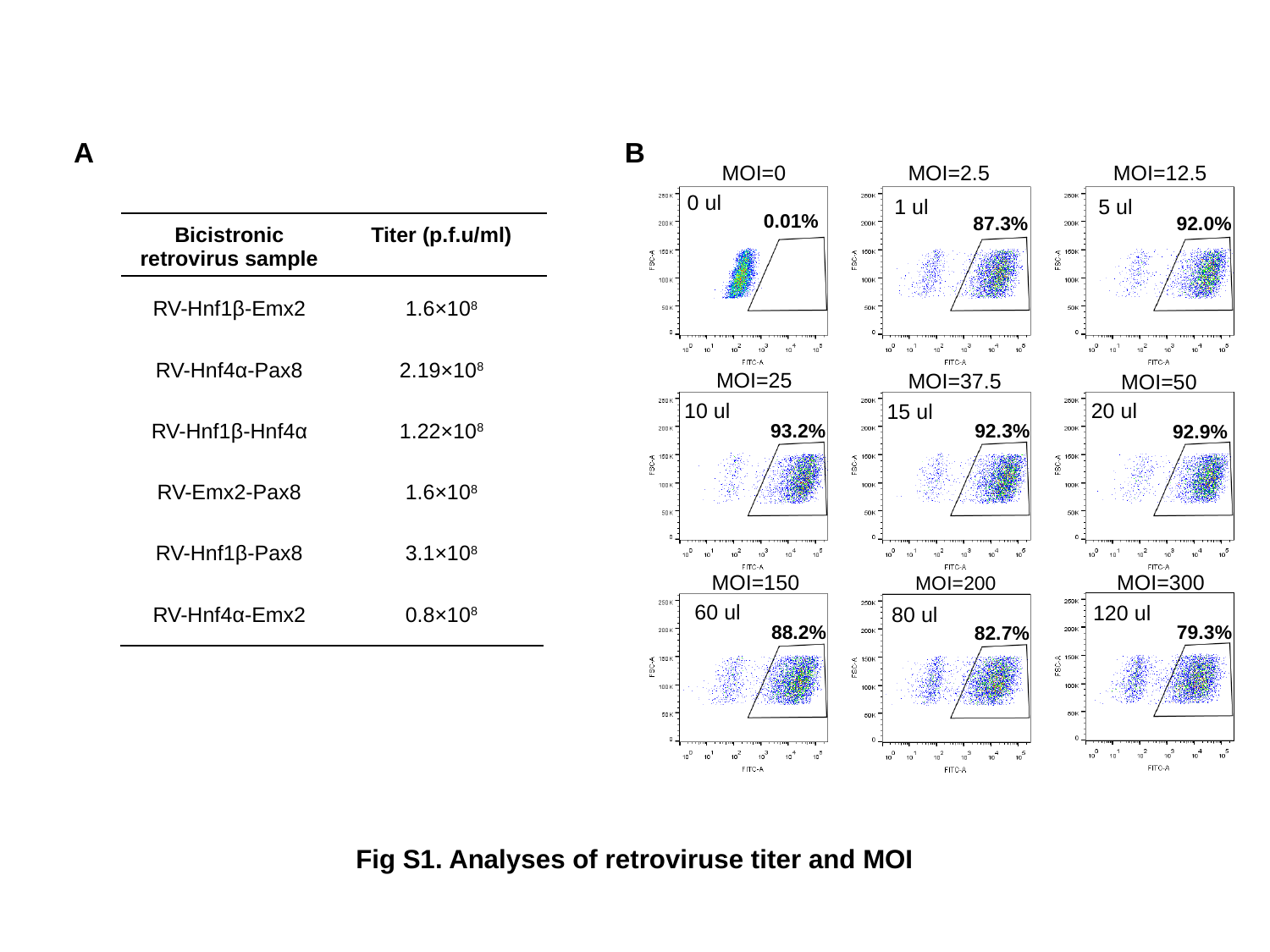

A
B
MOI=0
MOI=2.5
MOI=12.5
MOI=25
MOI=37.5
MOI=50
MOI=300
MOI=150
MOI=200
0 ul
1 ul
5 ul
0.01%
92.0%
87.3%
10 ul
20 ul
15 ul
93.2%
92.3%
92.9%
60 ul
120 ul
80 ul
79.3%
88.2%
82.7%
| |
| --- |
| Bicistronic retrovirus sample | Titer (p.f.u/ml) |
| --- | --- |
| RV-Hnf1β-Emx2 | 1.6×10^8^ |
| RV-Hnf4α-Pax8 | 2.19×10^8^ |
| RV-Hnf1β-Hnf4α | 1.22×10^8^ |
| RV-Emx2-Pax8 | 1.6×10^8^ |
| RV-Hnf1β-Pax8 | 3.1×10^8^ |
| RV-Hnf4α-Emx2 | 0.8×10^8^ |
| |
| --- |
Fig S1. Analyses of retroviruse titer and MOI

## Slide 2
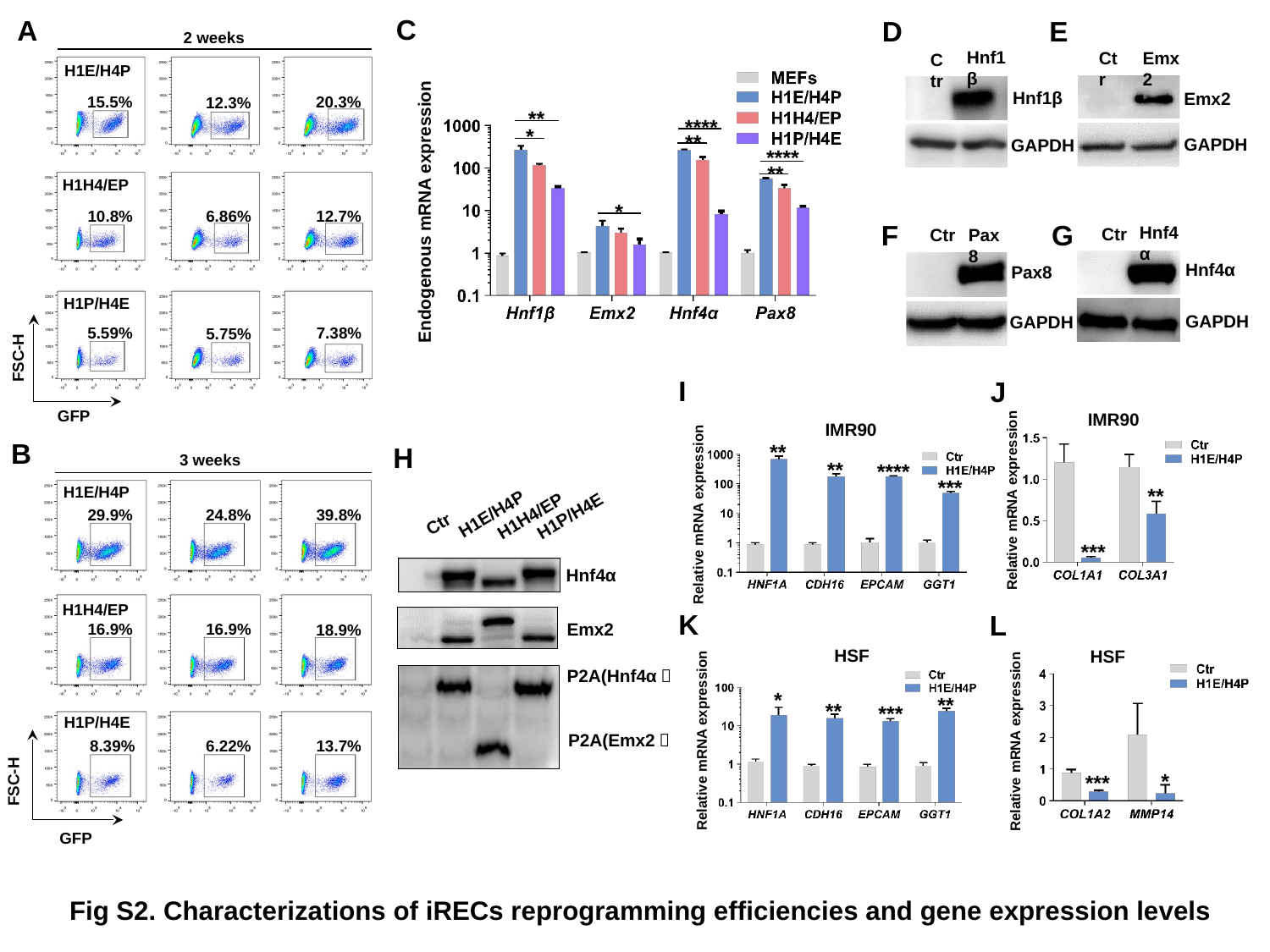

C
**
****
*
**
****
**
*
Endogenous mRNA expression
A
2 weeks
FSC-H
GFP
H1E/H4P
15.5%
12.3%
H1H4/EP
6.86%
12.7%
10.8%
H1P/H4E
7.38%
5.59%
5.75%
E
Emx2
Ctr
Emx2
GAPDH
D
Hnf1β
Ctr
Hnf1β
GAPDH
Ctr
Pax8
F
Pax8
GAPDH
Hnf4α
Ctr
G
Hnf4α
GAPDH
20.3%
I
J
**
IMR90
Relative mRNA expression
***
IMR90
Relative mRNA expression
B
3 weeks
H1E/H4P
H1H4/EP
H1P/H4E
29.9%
39.8%
24.8%
16.9%
16.9%
18.9%
8.39%
13.7%
6.22%
FSC-H
GFP
**
**
****
***
H
Ctr
H1E/H4P
H1H4/EP
H1P/H4E
Hnf4α
Emx2
P2A(Hnf4α）
P2A(Emx2）
K
HSF
Relative mRNA expression
*
**
**
***
L
HSF
Relative mRNA expression
*
***
Fig S2. Characterizations of iRECs reprogramming efficiencies and gene expression levels

## Slide 3
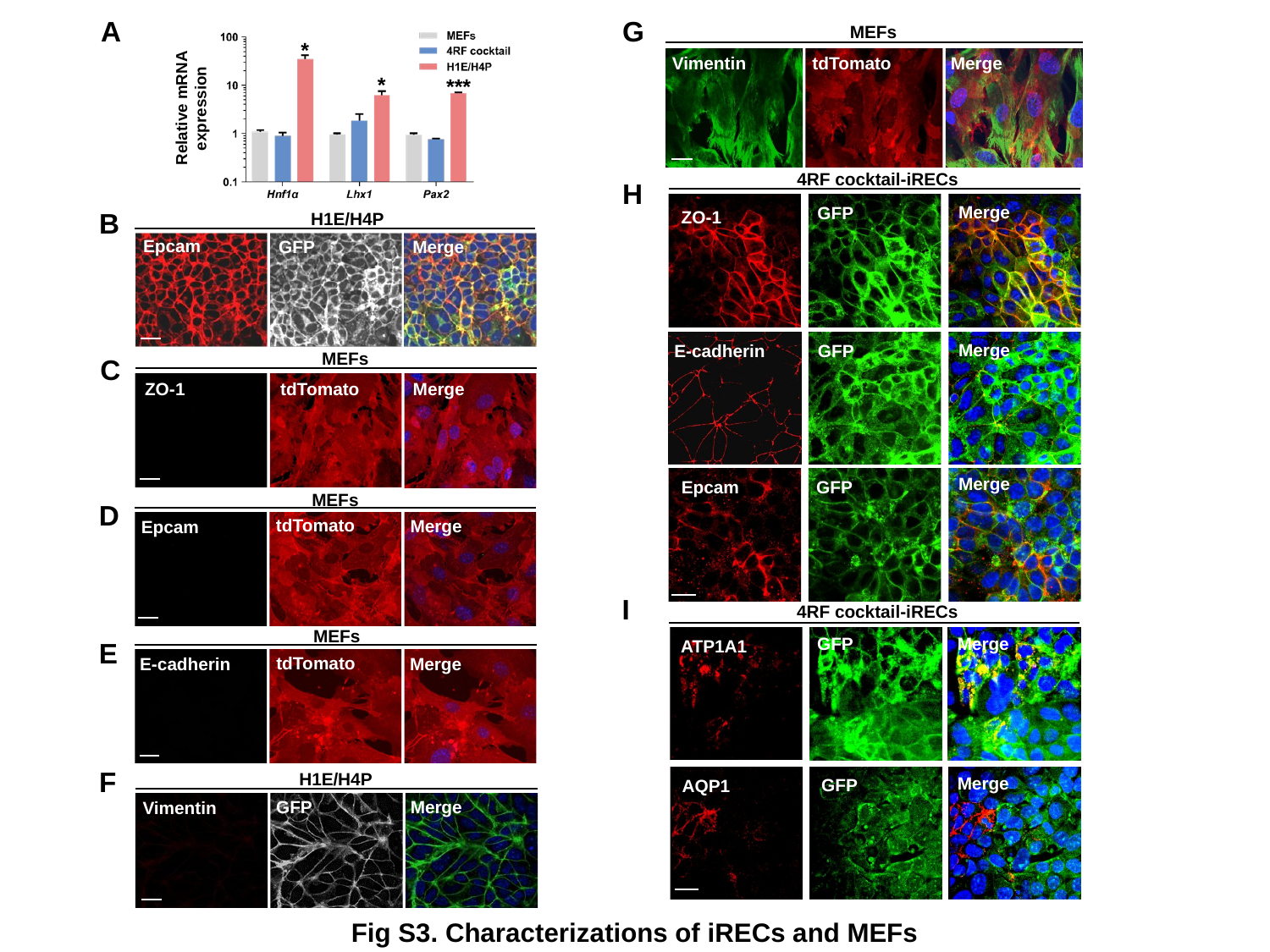

A
G
MEFs
Vimentin
tdTomato
Merge
*
*
***
Relative mRNA expression
4RF cocktail-iRECs
H
Merge
GFP
Merge
E-cadherin
GFP
Merge
GFP
Epcam
ZO-1
B
H1E/H4P
Epcam
GFP
Merge
MEFs
tdTomato
ZO-1
Merge
C
Epcam
MEFs
D
tdTomato
Merge
Epcam
MEFs
E
tdTomato
Merge
E-cadherin
I
4RF cocktail-iRECs
GFP
Merge
ATP1A1
Merge
GFP
AQP1
F
H1E/H4P
Merge
GFP
Vimentin
Fig S3. Characterizations of iRECs and MEFs

## Slide 4
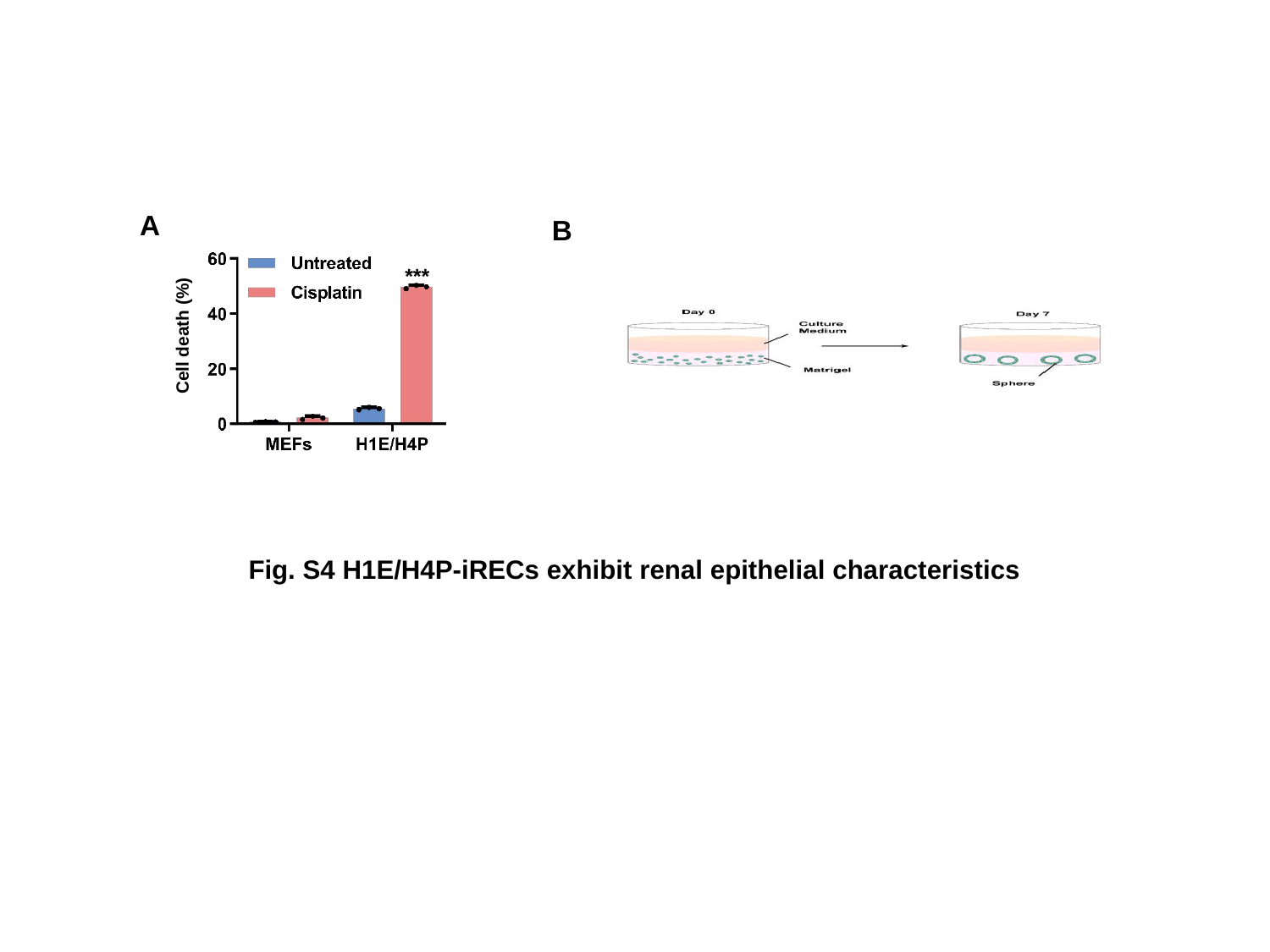

A
Cell death (%)
B
***
Fig. S4 H1E/H4P-iRECs exhibit renal epithelial characteristics
